# Diagnostic Performance of Ultrasonography and Mammography in Detecting Local Recurrence after Breast-conserving Surgery: A Systematic Review and Exploratory Diagnostic Meta-analysis

**DOI:** 10.2174/0115734056484401260820095303

**Published:** 2026-08-31

**Authors:** Chengcheng Yu, Hecheng Zhang, Haiyan Deng

**Affiliations:** 1 Department of Radiology, Beijing Hospital of Integrated Traditional Chinese and Western Medicine, Beijing, China; 2 Department of Cancer, Beijing Hospital of Integrated Traditional Chinese and Western Medicine, Beijing, China

**Keywords:** Breast cancer, Breast-conserving surgery, Local recurrence, Mammography, Ultrasonography, Diagnostic accuracy, HSROC

## Abstract

**Introduction/Background:**

Imaging surveillance after Breast-Conserving Surgery (BCS) is essential for identifying potentially curable local or ipsilateral breast tumor recurrence. Mammography (MG) remains the standard surveillance modality, but postoperative scarring, radiotherapy-related distortion, and breast density may reduce its interpretability. Ultrasonography (US) may provide complementary assessment of non-calcified soft-tissue abnormalities.

**Materials and Methods:**

A systematic search of PubMed, Embase, Web of Science, the Cochrane Library, and Ovid MEDLINE was performed from database inception to August 2025. Eligible studies enrolled patients after BCS or breast-conserving treatment, evaluated MG and/or US for postoperative local or ipsilateral recurrence, and reported extractable diagnostic accuracy data. Study selection, data extraction, and QUADAS-2 assessment were conducted independently by two reviewers. Owing to marked threshold and methodological heterogeneity, modality-specific performance was summarized using paired forest plots, Hierarchical Summary Receiver Operating Characteristic (HSROC) curves, Area Under the Curve (AUC), and leave-one-out sensitivity analyses rather than simple pooled sensitivity/specificity estimates.

**Results:**

Nine studies were included. Six studies contributed MG-specific data. MG sensitivity ranged from 0.25 to 1.00 and specificity from 0.51 to 1.00; the HSROC AUC was 0.752, and leave-one-out AUCs ranged from 0.732 to 0.839. Three studies contributed US-specific data. US sensitivity ranged from 0.50 to 0.95 and specificity was consistently high (approximately 0.98 in the included modality-specific studies); the HSROC AUC was 0.985, and leave-one-out AUCs ranged from 0.966 to 0.985. Because direct comparative evidence was limited and heterogeneous, no formal pooled superiority estimate was retained.

**Discussion:**

The available evidence suggests that US may be a valuable complementary modality after BCS, particularly for soft-tissue or non-calcified recurrence, whereas MG remains important for detecting microcalcifications and architectural changes. The evidence base is limited by small numbers of studies, variable reference standards, older imaging technology in some reports, and incomplete reporting of recurrence morphology and surveillance timing.

**Conclusion:**

US appears to have complementary value in postoperative surveillance after BCS, but the current evidence does not justify replacing MG or making an unconditional claim that US is superior. Larger prospective, head-to-head studies using standardized imaging protocols and reference standards are warranted.

## 1 INTRODUCTION

Breast-Conserving Surgery (BCS) followed by radiotherapy is an established treatment for many patients with early breast cancer and can achieve oncologic outcomes comparable to mastectomy while preserving breast anatomy and quality of life [1-4]. Nevertheless, ipsilateral breast tumor recurrence remains clinically important because early detection may enable salvage treatment and improve long-term outcomes [5-7].

Mammography (MG) is the conventional basis of post-BCS imaging surveillance. It is particularly useful for detecting microcalcifications, architectural distortion, and other mammographic signs of recurrent malignancy [8-10]. However, the treated breast is often difficult to interpret because surgical scars, edema, fat necrosis, radiotherapy-related parenchymal changes, and postoperative architectural distortion may either obscure a small recurrence or mimic malignancy [9, 10].

Ultrasonography (US) provides real-time, radiation-free evaluation of soft-tissue structures and can help characterize palpable or mammographically occult masses. It may be especially useful for dense breasts and for non-calcified recurrent lesions that are less conspicuous on mammography [11, 12]. However, US is operator-dependent and may generate false-positive findings in a postoperative breast, especially when scar tissue and fat necrosis are present.

Previous reviews of surveillance imaging after primary breast cancer treatment have emphasized the importance of mammography but have also noted uncertainty regarding the incremental value of US, MRI, and other modalities [13-15]. Directly comparing US and MG after BCS is therefore clinically relevant but methodologically challenging because studies differ in patient selection, imaging protocol, diagnostic threshold, reference standard, timing of follow-up, and the morphology of recurrence. This systematic review and exploratory diagnostic meta-analysis reassessed the diagnostic performance of MG and US for detecting local or ipsilateral recurrence after BCS, with special attention to heterogeneity and limitations of the available evidence.

## 2 MATERIALS AND METHODS

### 2.1 Reporting Guideline and Protocol Registration

This review was prepared in accordance with the PRISMA 2020 statement and the principles of PRISMA-DTA for diagnostic test accuracy reviews [16, 17]. The review protocol was not prospectively registered in PROSPERO or another registry. The absence of prospective registration is acknowledged as a methodological limitation because it reduces transparency and increases the risk of selective reporting.

### 2.2 Literature Search

A systematic search was conducted across five English-language databases: PubMed, Embase, Web of Science, the Cochrane Library, and Ovid MEDLINE. The search covered database inception to August 2025 and was restricted to studies published in English. The search strategy was structured according to the PICOS framework and combined controlled vocabulary terms with free-text terms for breast cancer, BCS or breast-conserving treatment, mammography, ultrasonography, MRI, and local or ipsilateral recurrence. The complete PubMed strategy and database-specific search terms are provided in Table **S1**.

### 2.3 Eligibility Criteria

The inclusion criteria were: (1) patients after BCS or breast-conserving treatment for breast cancer; (2) MG and/or US evaluated as an index test, with extractable data for MG alone, US alone, or combined MG plus US; (3) the target condition was postoperative local or ipsilateral breast tumor recurrence; (4) the study reported a 2 x 2 diagnostic table or data from which sensitivity, specificity, True Positives (TP), False Positives (FP), False Negatives (FN), and True Negatives (TN) could be extracted or recalculated; and (5) original clinical research, including prospective cohort, retrospective cohort, case-control, or cross-sectional diagnostic accuracy studies.

The exclusion criteria were: (1) reviews, systematic reviews, meta-analyses, commentaries, case reports, conference abstracts, and non-original publications; (2) studies reporting only distant metastasis, axillary recurrence, contralateral breast cancer, or overall recurrence without separable ipsilateral local recurrence data; (3) studies without extractable or calculable sensitivity and specificity; (4) duplicate reports from the same cohort, for which the most complete report was retained; (5) unavailable full text or severely incomplete key data; and (6) non-human or non-English studies. A formal PICOS summary is provided in Table **S2**.

### 2.4 Study Selection and Data Extraction

Two reviewers (C.Y., H.Z.) independently screened titles and abstracts and then assessed full texts according to the predefined criteria. Disagreements were resolved by discussion and, when necessary, by a third reviewer (H.D.). Reasons for exclusion were recorded during full-text screening.

Two reviewers (C.Y., H.Z.) independently extracted study characteristics, patient characteristics, surgery type, reconstruction status, imaging modality and protocol, surveillance interval, recurrence definition, reference standard, and 2x2 diagnostic data. When key data were unclear, the corresponding authors were contacted when possible. For studies reporting multiple mammographic approaches, conventional MG or Digital Mammography (DM) alone was used for the main MG analysis to maintain consistency. Studies reporting only combined or non-separable modality results were described qualitatively and were not entered into modality-specific HSROC synthesis.

### 2.5 Quality Assessment

Methodological quality and applicability were assessed using QUADAS-2, which evaluates patient selection, index test, reference standard, and flow and timing domains [18]. Each domain was rated as low, high, or unclear risk of bias; applicability concerns were rated separately. The results were summarized using a traffic-light plot.

### 2.6 Statistical Analysis

All analyses were performed using R version 4.4.0. MG-related and US-related examinations were analyzed separately. Exact binomial 95% confidence intervals were calculated for each study's sensitivity and specificity. Paired forest plots were used to display study-level sensitivity and specificity and to visualize between-study variability.

Because the included studies differed in diagnostic threshold, imaging protocol, recurrence definition, and reference standard, simple pooling of sensitivity and specificity at a single threshold was considered inappropriate. Instead, HSROC curves were fitted separately for MG and US using the mada package, and the AUC was used as an exploratory summary of overall diagnostic discrimination across thresholds. Leave-one-out sensitivity analyses were performed by excluding one study at a time and refitting the HSROC model. The previous draft's direct OR-based comparison was removed because an unadjusted odds ratio is not an appropriate primary effect measure for diagnostic accuracy meta-analysis. Formal publication-bias testing was not performed because fewer than ten studies were available in each modality-specific subgroup, making funnel-plot asymmetry tests underpowered and potentially misleading.

## 3 RESULTS

### 3.1 Literature Selection

The database search identified 1,398 records: PubMed (n = 545), Embase (n = 499), Web of Science (n = 3), the Cochrane Library (n = 13), and Ovid MEDLINE (n = 338). After removal of 490 duplicates, 908 records underwent title and abstract screening. A total of 871 records were excluded, leaving 37 reports for retrieval. Five full texts were unavailable, and 32 reports were assessed for eligibility. Twenty-three reports were excluded because of an ineligible population (n = 1), inappropriate index test (n = 3), absence of relevant diagnostic accuracy outcomes (n = 6), or insufficient/non-extractable data (n = 13). Ultimately, nine studies were included in the systematic review [19-27]. The study selection process is shown in Fig. (**1**).

### 3.2 Study Characteristics

The nine included studies were published between 1988 and 2024 and were conducted in Germany, Scotland, Japan, South Korea, Egypt, Thailand, and South Africa. Most studies were retrospective, whereas one was prospective. Surgical approaches included BCS, breast-conserving treatment, lumpectomy, quadrantectomy, oncoplastic BCS, and selected reconstructive procedures. The imaging modalities included MG or DM, US, DBT, AI-CAD, abbreviated MRI, and selective MRI. Table **1** summarizes the baseline characteristics and reference standards of the included studies.

### 3.3 Quality Assessment

Most studies were rated as low risk in the index test and reference standard domains. Some concerns were noted in patient selection, especially in older or retrospective studies, and in flow and timing for studies in which not all patients received the same verification pathway. Applicability concerns were generally low because the populations and index tests were aligned with postoperative surveillance after BCS. The QUADAS-2 methodological quality assessment is shown in Fig. (**2**).

### 3.4 Diagnostic Accuracy of Mammography-related Examinations

Six studies contributed MG-specific diagnostic data (Table **2**). The paired forest plot (Fig. **3**) showed substantial variation in sensitivity across studies, ranging from 0.25 to 1.00. The lowest sensitivity estimates were observed in An *et al*. [19] and Kim *et al*. [21], whereas Sondezi *et al*. [27] reported the highest sensitivity. Specificity ranged from 0.51 to 1.00; Mansour *et al*. [25] had the lowest specificity, reflecting the high number of false-positive postoperative mammographic findings in that cohort. HSROC analysis showed that mammography-related examinations had an AUC of 0.752 across different diagnostic thresholds (Fig. **4**), indicating moderate diagnostic discrimination. Leave-one-out sensitivity analysis showed that the MG AUC ranged from 0.732 to 0.839 after sequential exclusion of individual studies (Fig. **5**). Excluding Mansour *et al*. [25] increased the AUC to 0.839, suggesting that the study's low specificity significantly influenced the overall curve.

### 3.5 Diagnostic Accuracy of Ultrasound-related Examinations

Three studies contributed US-specific diagnostic data (Table **2**). The paired forest plot (Fig. **6**) showed consistently high specificity across studies, with estimates close to 0.98. Sensitivity varied from 0.50 to 0.95. Kubota *et al*. [22] reported the highest sensitivity. In contrast, Kim *et al*. [21] and An *et al*. [19] both reported sensitivities of approximately 0.50, with wide confidence intervals, reflecting the small number of recurrence cases. HSROC analysis showed that study points for ultrasound-related examinations were mainly distributed in the low false-positive-rate region, with an AUC of 0.985 (Fig. **7**), suggesting high overall discrimination in the available US-specific evidence. Leave-one-out sensitivity analysis showed AUC values ranging from 0.966 to 0.985 after excluding one study at a time (Fig. **8**), indicating relative stability of the exploratory synthesis. However, this finding should be interpreted cautiously because only three modality-specific US studies were available.

### 3.6 Direct Comparison and Clinical Synthesis

The previous direct comparison based on a pooled odds ratio was removed. Only a small subset of studies provided head-to-head MG and US data, and the clinical and statistical heterogeneity was substantial. Therefore, the revised analysis does not claim statistically proven superiority of one modality over the other. Instead, the findings support a clinically complementary interpretation: MG remains important for calcifications and architectural distortion, whereas US may improve evaluation of soft-tissue or non-calcified abnormalities in the treated breast.

## 4 DISCUSSION

This systematic review shows that both MG and US have diagnostic value for postoperative local or ipsilateral recurrence after BCS, but the strength of inference differs by modality and is limited by the available evidence. MG-related examinations showed moderate HSROC discrimination, with considerable variation in sensitivity and specificity. US-related examinations showed high specificity and high HSROC discrimination in the available studies, but only three studies contributed modality-specific US data and sensitivity estimates were imprecise in studies with few recurrent events.

The clinical difference between MG and US should not be reduced to a simple statement that one modality is universally better. MG remains essential in post-BCS surveillance because recurrent ductal carcinoma *in situ* or invasive recurrence can manifest as suspicious microcalcifications, architectural distortion, or evolving density, which may be more readily detected on mammography [8-10]. Conversely, US can provide targeted assessment of the surgical bed, palpable findings, and non-calcified soft-tissue lesions, and may be especially valuable in dense breasts or when postoperative distortion obscures the mammographic background [11, 12].

The high specificity of US in the modality-specific studies suggests that US may help exclude recurrence when benign postoperative changes are well characterized. However, US sensitivity varied substantially, and wide confidence intervals in small studies indicate that uncertainty remains. Operator dependence, variable BI-RADS thresholds, differences in radiologist experience, breast density, oncoplastic material, follow-up interval, and recurrence morphology may all contribute to between-study heterogeneity.

MRI was not analyzed as an index test in the primary quantitative synthesis because the objective of this review was to compare MG and US, and MRI data were not consistently available across the included studies. Nevertheless, MRI is clinically relevant in selected post-treatment settings. Previous evidence suggests that MRI can be highly sensitive for detecting further breast cancer after primary treatment, but its use is constrained by cost, availability, contrast administration, and false-positive findings [14, 15]. Therefore, MRI should be considered an adjunctive option for selected high-risk patients or for equivocal findings, rather than a universal replacement for MG or US.

The results are broadly consistent with the existing literature that imaging surveillance after breast cancer treatment remains complex and that evidence for supplemental modalities is heterogeneous [13-15]. Compared with the previous version of the manuscript, the revised analysis avoids overinterpreting pooled sensitivity and specificity under extreme heterogeneity, removes the inappropriate OR-based direct comparison, and frames the evidence as exploratory.

## 5 STUDY LIMITATIONS

First, the review protocol was not prospectively registered, which may reduce transparency and should be considered when interpreting the findings. Second, only nine studies met the eligibility criteria, and fewer studies contributed modality-specific 2 x 2 data for the main quantitative synthesis. Third, some included studies were retrospective and used different verification pathways; suspicious findings were usually confirmed histologically, whereas negative or benign findings often relied on clinical or imaging follow-up. Fourth, the included studies differed in imaging technology, diagnostic thresholds, follow-up intervals, surgical techniques, reconstruction status, and recurrence morphology. Fifth, some clinically important subgroup analyses, including radiotherapy status, calcified *versus* mass-like recurrence, breast density, and time from surgery to surveillance, could not be performed because these variables were not consistently reported. Sixth, one older study reflected historical imaging technology and was therefore not considered strong evidence for current practice. Finally, because fewer than ten studies were available in each modality-specific subgroup, formal publication bias testing was not performed.

## CONCLUSION

US may provide clinically useful complementary information for detecting local or ipsilateral recurrence after BCS, particularly for soft-tissue or non-calcified abnormalities. MG remains an important surveillance modality, especially for detecting microcalcifications and architectural distortion. Given the limited number of modality-specific studies, variable reference standards, and substantial heterogeneity, the present evidence does not support an unconditional conclusion that US is superior to MG. Future prospective, multicenter, head-to-head studies should use standardized imaging protocols, predefined positivity thresholds, uniform reference standards, and stratified reporting by recurrence morphology and surveillance interval.

## Figures and Tables

**Fig. (1):**
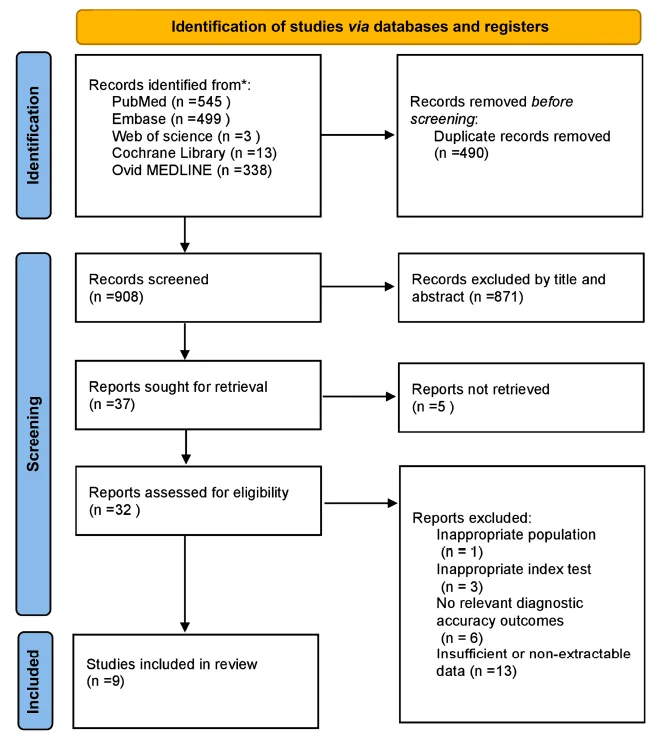
PRISMA flow diagram for study identification, screening, eligibility assessment, and inclusion.

**Fig. (2):**
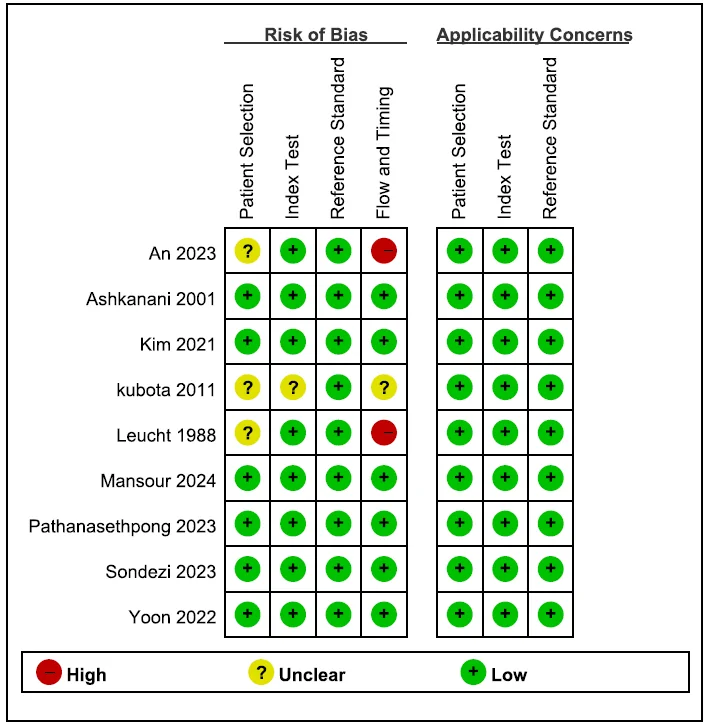
QUADAS-2 methodological quality assessment.

**Fig. (3):**
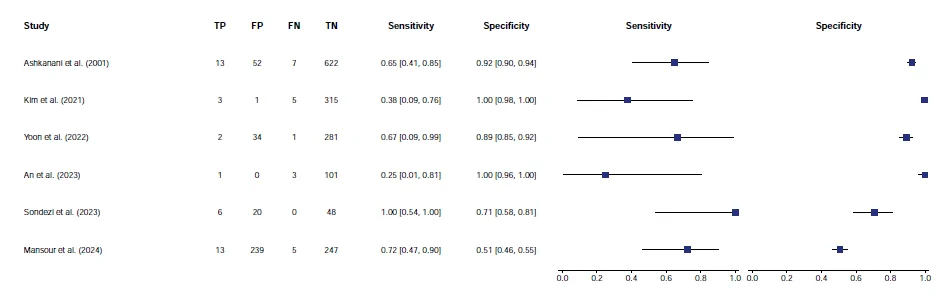
Forest plot of the diagnostic accuracy of mammography for local recurrence after breast-conserving surgery.

**Fig. (4):**
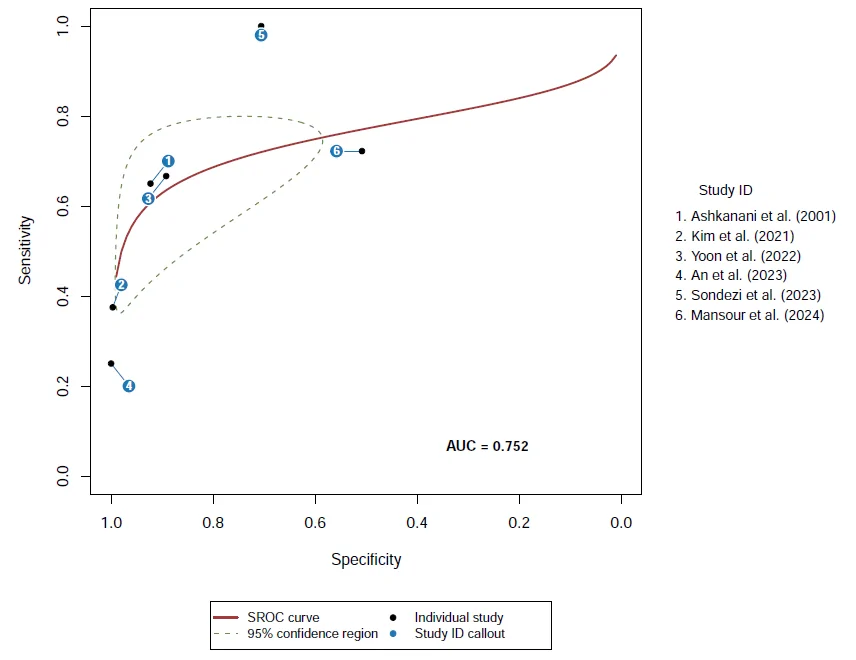
SROC curve of mammography for recurrence after breast-conserving surgery.

**Fig. (5):**
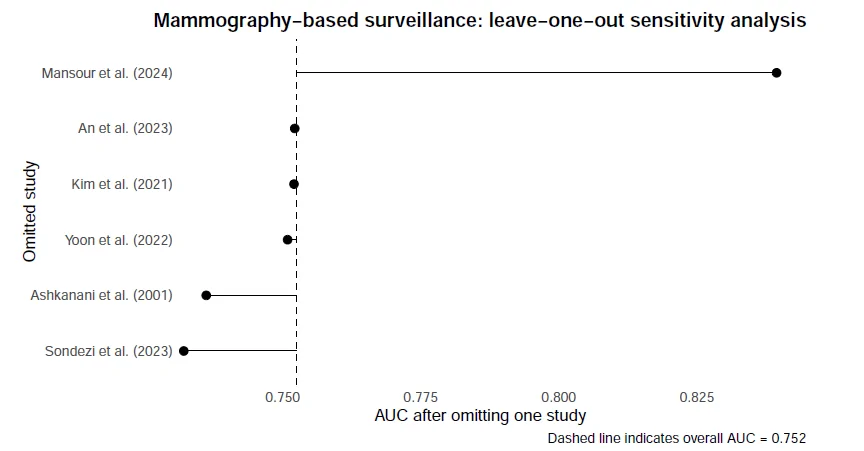
Sensitivity analysis of mammography for recurrence after breast-conserving surgery.

**Fig. (6):**
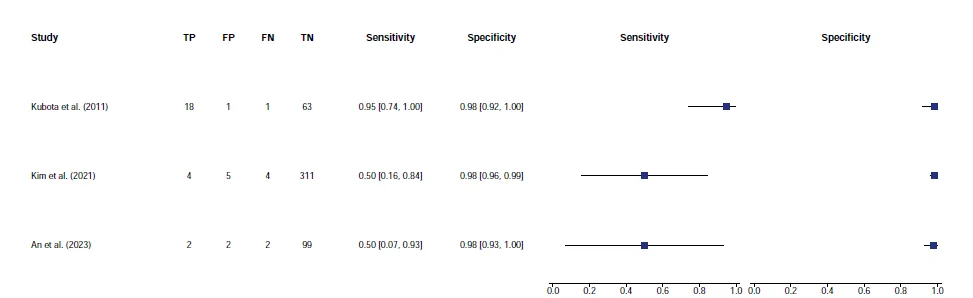
Forest plot of the diagnostic accuracy of breast ultrasound for local recurrence after breast-conserving surgery.

**Fig. (7):**
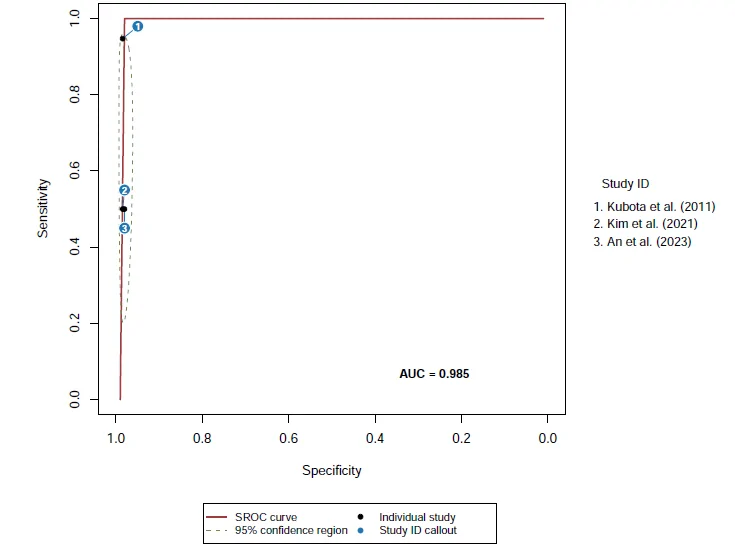
SROC curve of breast ultrasound for recurrence after breast-conserving surgery.

**Fig. (8):**
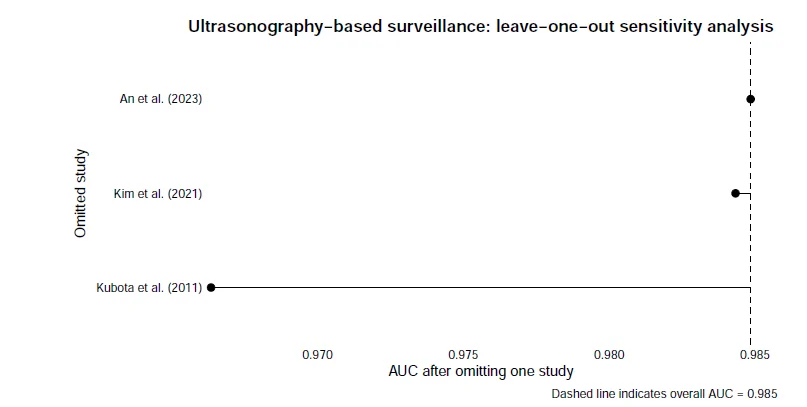
Sensitivity analysis of breast ultrasound for recurrence after breast-conserving surgery.

**Table 1:** Baseline characteristics of the included studies.

**Study/Refs.**	**Sample Size**	**Patient Age, Years (Mean/Median (range))**	**Design**	**Reference Standard**	**Surgery/Reconstruction**	**Follow-up or Exam Frequency**	**Imaging Methods**
Leucht & Rabe (1988, Germany) [24]	106	Mean 51 (27–78)	Retrospective	Biopsy	Breast-conserving surgery; no reconstruction	Not reported	US + MG
Ashkanani **et al*. * (2001, Scotland) [23]	695	Mean 59 (33–76)	Prospective	FNAC or biopsy	Breast-conservation surgery; no reconstruction	Follow-up 3.5 years (2-7 years)	MG
Kubota *et al* . (2011, Japan) [22]	272	Not specified; patients with early breast cancer	Retrospective	Core needle biopsy, aspiration cytology, and/or long-term ultrasound follow-up	Breast-conserving therapy	Every 6 months for first 8 years, then annually	US
Yoon **et al*. * (2022, South Korea) [20]	314	Mean 53.3 ± 10.6 (SD)	Retrospective	Pathology from biopsy/surgery	Breast-conserving therapy; no reconstruction	Not specified	DM; DM + DBT; DM + AI-CAD
Kim **et al*. * (2021, South Korea) [21]	324	Mean 53.9 years (22–83)	Retrospective	Biopsy	Oncoplastic BCS with acellular dermal matrix	MG and US every 6 months for first 2 years, then annually	MG; US; abbreviated MRI
An **et al*. * (2023, South Korea) [19]	105	Mean 51.9 (25–75)	Retrospective	Pathological tissue diagnosis or at least 2-year follow-up	Oncoplastic BCS with acellular dermal matrix	MG and US every 6 months for first 2 years, then annually	MG; US; selective MRI
Mansour *et al* . (2024, Egypt) [25]	577	Mean 43.89 ± 5.99 (SD)	Retrospective	Biopsy for suspicious/malignant findings; ultrasound plus 1-year follow-up for benign findings	Breast conservation (lumpectomy, quadrantectomy, oncoplastic) and reconstruction	Not specified	Digital mammography + AI-assisted reading + US
Pathanasethpong **et al*. * (2023, Thailand) [26]	130	Median 49.75 (27–70)	Retrospective	Pathological report or 5-year imaging follow-up	Breast-conserving treatment	4-12 months postoperative follow-up	MG; US
Sondezi *et al* . (2023, South Africa) [27]	92	Mean 54.3 (31–79)	Retrospective	Histopathology and >=5-year clinical/radiological follow-up	Breast-conserving therapy	At least 5 years	US; MG; MRI in selected cases

**Table 2:** Modality-specific 2 x 2 diagnostic data used in the exploratory synthesis.

**Modality**	**Study**	**TP**	**FP**	**FN**	**TN**	**N analyzed**	**Sensitivity (95% CI)**	**Specificity (95% CI)**	**Notes**
MG	An **et al*. * (2023) [19]	1	0	3	101	105	0.25 (0.01-0.81)	1.00 (0.96-1.00)	Conventional MG
MG	Kim **et al*. * (2021) [21]	3	0	5	316	324	0.38 (0.09-0.76)	1.00 (0.99-1.00)	Conventional MG
MG	Ashkanani **et al*. * (2001) [23]	13	54	7	621	695	0.65 (0.41-0.85)	0.92 (0.90-0.94)	MG surveillance
MG	Yoon **et al*. * (2022) [20]	4	33	2	275	314	0.67 (0.22-0.96)	0.89 (0.85-0.93)	DM alone
MG	Mansour **et al*. * (2024) [25]	13	239	5	247	504	0.72 (0.47-0.90)	0.51 (0.46-0.55)	DM alone; analyzed examinations
MG	Sondezi **et al*. * (2023) [27]	6	25	0	61	92	1.00 (0.54-1.00)	0.71 (0.60-0.80)	MG during follow-up
US	Kubota **et al*. * (2011) [22]	18	5	1	248	272	0.95 (0.74-1.00)	0.98 (0.95-0.99)	BI-RADS US
US	Kim **et al*. * (2021) [21]	4	6	4	310	324	0.50 (0.16-0.84)	0.98 (0.96-0.99)	US
US	An **et al*. * (2023) [19]	2	2	2	99	105	0.50 (0.07-0.93)	0.98 (0.93-1.00)	US

**Abbreviations:** TP = true positive; FP = false positive; FN = false negative; TN = true negative. Counts were extracted or recalculated from the reported diagnostic indices and denominators in the included studies. For studies with multiple mammographic approaches, the conventional MG/DM-alone result was used for the main analysis.

## Data Availability

All data analyzed in this review were extracted from published studies. The extracted diagnostic accuracy dataset used for the exploratory synthesis is summarized in Table **2**.

## LIST OF ABBREVIATIONS

BCS: Breast-conserving surgery
BCT: Breast-conserving treatment
MG: Mammography
DM: Digital mammography
US: Ultrasonography
MRI: Magnetic resonance imaging
DBT: Digital breast tomosynthesis
AI-CAD: Artificial intelligence-based computer-aided detection
HSROC: Hierarchical summary receiver operating characteristic
SROC: Summary receiver operating characteristic
AUC: Area under the curve
QUADAS-2: Quality Assessment of Diagnostic Accuracy Studies 2
PRISMA: Preferred Reporting Items for Systematic Reviews and Meta-Analyses
PRISMA-DTA: Preferred Reporting Items for a Systematic Review and Meta-analysis of Diagnostic Test Accuracy Studies
TP: True positive
FP: False positive
FN: False negative
TN: True negative
FNAC: Fine-needle aspiration cytology
BI-RADS: Breast Imaging Reporting and Data System

## STANDARDS OF REPORTING

PRISMA and PRISMA-DTA guidelines and methodology were followed.

## SUPPLEMENTRY MATERIAL

PRISMA checklist is available as supplementary material on the publisher’s website along with the published article. Supplementary material is available on the publisher’s website along with the published article.
