# Supplementary material for: Diagnostic Performance of Ultrasonography and Mammography in Detecting Local Recurrence after Breast-conserving Surgery: A Systematic Review and Exploratory Diagnostic Meta-analysis
Source: Curr Med Imaging. 2026 Aug 31;22:E15734056484401. doi: 10.2174/0115734056484401260820095303 (PMC13741129; doi:10.2174/0115734056484401260820095303)
Supplement: Supplementary file 1 [file CMIM-22-E15734056484401_SD1.pdf]

1875-6603/26 *Send Orders for Reprints to reprints@benthamscience.net*

BENTHAM SCIENCE
CrossMark

Current Medical Imaging

Content list available at: https://benthamscience.com/journals/cmir

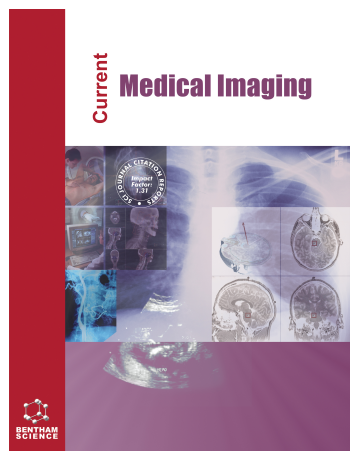

Supplementary Material

Diagnostic Performance of Ultrasonography and Mammography in Detecting Local Recurrence after Breast-Conserving Surgery: A Systematic Review and Exploratory Diagnostic Meta-analysis

Chengcheng Yu[1], Hecheng Zhang[1] and Haiyan Deng[2,*]

[1]*Department of Radiology, Beijing Hospital of Integrated Traditional Chinese and Western Medicine, Beijing, China*
[2]*Department of Cancer, Beijing Hospital of Integrated Traditional Chinese and Western Medicine, Beijing, China*

Table S1. Search strategy used for the systematic review.

Item	Search details
PICOS element	Terms
Population	"breast cancer" OR "breast neoplasm" OR "breast carcinoma"
Surgery/context	"breast-conserving surgery" OR "breast conserving surgery" OR "breast-conserving therapy" OR "breast conserving treatment" OR lumpectomy OR quadrantectomy
Index tests	mammography OR mammogram OR "digital mammography" OR tomosynthesis OR ultrasonography OR ultrasound OR sonography OR "breast ultrasound" OR MRI OR "magnetic resonance imaging"
Target condition	recurrence OR "local recurrence" OR "ipsilateral breast tumor recurrence" OR "ipsilateral recurrence" OR "postoperative recurrence" OR surveillance
PubMed example	(("Breast Neoplasms"[Mesh] OR breast cancer OR breast carcinoma) AND ("breast-conserving surgery" OR "breast conserving therapy" OR lumpectomy) AND (mammography OR "Mammography"[Mesh] OR ultrasonography OR "Ultrasonography, Mammary"[Mesh] OR ultrasound OR MRI OR "Magnetic Resonance Imaging"[Mesh]) AND (recurrence OR "local recurrence" OR "ipsilateral breast tumor recurrence" OR surveillance))

Table S2. PICOS framework and eligibility criteria.

PICOS domain	Definition
Population	Patients after BCS or breast-conserving treatment for breast cancer
Index test	MG/DM and/or US; studies with MRI, DBT, or AI-CAD included only if MG or US data were separable
Comparator	Alternative imaging modality, combined imaging, or reference standard; head-to-head comparison not required
Outcome	Local or ipsilateral breast tumor recurrence with extractable diagnostic accuracy data
Study design	Original clinical diagnostic accuracy studies, including prospective or retrospective cohorts, case-control, or cross-sectional studies
Main exclusions	Reviews, case reports, conference abstracts, non-human studies, non-English studies, unavailable full text, duplicate cohorts, non-local recurrence outcomes, and non-extractable diagnostic data

DOI: 10.2174/0115734056484401260820095303, 2026, *22*, e15734056484401

PRISMA 2020 Checklist.

Section and Topic	Item #	Checklist item	Location where item is reported
**TITLE**			
Title	1	Identify the report as a systematic review.	Title page.
**ABSTRACT**			
Abstract	2	See the PRISMA 2020 for Abstracts checklist.	Abstract.
**INTRODUCTION**			
Rationale	3	Describe the rationale for the review in the context of existing knowledge.	Introduction.
Objectives	4	Provide an explicit statement of the objective(s) or question(s) the review addresses.	Introduction.
**METHODS**			
Eligibility criteria	5	Specify the inclusion and exclusion criteria for the review and how studies were grouped for the syntheses.	Methods, Section 2.3.
Information sources	6	Specify all databases, registers, websites, organisations, reference lists and other sources searched or consulted to identify studies. Specify the date when each source was last searched or consulted.	Methods, Section 2.2.
Search strategy	7	Present the full search strategies for all databases, registers and websites, including any filters and limits used.	Methods, Section 2.2.
Selection process	8	Specify the methods used to decide whether a study met the inclusion criteria of the review, including how many reviewers screened each record and each report retrieved, whether they worked independently, and if applicable, details of automation tools used in the process.	Methods, Section 2.4.
Data collection process	9	Specify the methods used to collect data from reports, including how many reviewers collected data from each report, whether they worked independently, any processes for obtaining or confirming data from study investigators, and if applicable, details of automation tools used in the process.	Methods, Section 2.4.
Data items	10a	List and define all outcomes for which data were sought. Specify whether all results that were compatible with each outcome domain in each study were sought (e.g. for all measures, time points, analyses), and if not, the methods used to decide which results to collect.	Methods, Sections 2.3-2.4.
	10b	List and define all other variables for which data were sought (e.g. participant and intervention characteristics, funding sources). Describe any assumptions made about any missing or unclear information.	Methods, Section 2.4.
Study risk of bias assessment	11	Specify the methods used to assess risk of bias in the included studies, including details of the tool(s) used, how many reviewers assessed each study and whether they worked independently, and if applicable, details of automation tools used in the process.	Methods, Section 2.5.
Effect measures	12	Specify for each outcome the effect measure(s) (e.g. risk ratio, mean difference) used in the synthesis or presentation of results.	Methods, Section 2.6.
Synthesis methods	13a	Describe the processes used to decide which studies were eligible for each synthesis (e.g. tabulating the study intervention characteristics and comparing against the planned groups for each synthesis (item #5)).	Methods, Sections 2.3-2.6.
	13b	Describe any methods required to prepare the data for presentation or synthesis, such as handling of missing summary statistics, or data conversions.	Methods, Sections 2.4 and 2.6.
	13c	Describe any methods used to tabulate or visually display results of individual studies and syntheses.	Methods, Section 2.6.
	13d	Describe any methods used to synthesize results and provide a rationale for the choice(s). If meta-analysis was performed, describe the model(s), method(s) to identify the presence and extent of statistical heterogeneity, and software package(s) used.	Methods, Section 2.6.
	13e	Describe any methods used to explore possible causes of heterogeneity among study results (e.g. subgroup analysis, meta-regression).	Methods, Section 2.6.
	13f	Describe any sensitivity analyses conducted to assess robustness of the synthesized results.	Methods, Section 2.6.

Section and Topic	Item #	Checklist item	Location where item is reported
Reporting bias assessment	14	Describe any methods used to assess risk of bias due to missing results in a synthesis (arising from reporting biases).	Methods, Section 2.6.
Certainty assessment	15	Describe any methods used to assess certainty (or confidence) in the body of evidence for an outcome.	Not formally assessed. limitations of the evidence are discussed in Section 6.
**RESULTS**			
Study selection	16a	Describe the results of the search and selection process, from the number of records identified in the search to the number of studies included in the review, ideally using a flow diagram.	Results, Section 3.1 and Figure 1.
	16b	Cite studies that might appear to meet the inclusion criteria, but which were excluded, and explain why they were excluded.	Results, Section 3.1 and Figure 1.
Study characteristics	17	Cite each included study and present its characteristics.	Results, Section 3.2 and Table 1.
Risk of bias in studies	18	Present assessments of risk of bias for each included study.	Results, Section 3.3 and Figure 2.
Results of individual studies	19	For all outcomes, present, for each study: (a) summary statistics for each group (where appropriate) and (b) an effect estimate and its precision (e.g. confidence/credible interval), ideally using structured tables or plots.	Results, Sections 3.4-3.5; Figures 3 and 6; Table 2.
Results of syntheses	20a	For each synthesis, briefly summarise the characteristics and risk of bias among contributing studies.	Results, Sections 3.4-3.6.
	20b	Present results of all statistical syntheses conducted. If meta-analysis was done, present for each the summary estimate and its precision (e.g. confidence/credible interval) and measures of statistical heterogeneity. If comparing groups, describe the direction of the effect.	Results, Sections 3.4-3.5.
	20c	Present results of all investigations of possible causes of heterogeneity among study results.	Results, Sections 3.4-3.5; Discussion.
	20d	Present results of all sensitivity analyses conducted to assess the robustness of the synthesized results.	Results, Sections 3.4-3.5 and Figures 5 and 8.
Reporting biases	21	Present assessments of risk of bias due to missing results (arising from reporting biases) for each synthesis assessed.	Not performed; rationale reported in Methods, Section 2.6, and Limitations.
Certainty of evidence	22	Present assessments of certainty (or confidence) in the body of evidence for each outcome assessed.	Not formally assessed. The limitations and strength of inference are discussed in Discussion and Limitations.
**DISCUSSION**			
Discussion	23a	Provide a general interpretation of the results in the context of other evidence.	Discussion.
	23b	Discuss any limitations of the evidence included in the review.	Discussion; Limitations.
	23c	Discuss any limitations of the review processes used.	Limitations.
	23d	Discuss implications of the results for practice, policy, and future research.	Discussion; Conclusion; Limitations.
**OTHER INFORMATION**			
Registration and protocol	24a	Provide registration information for the review, including register name and registration number, or state that the review was not registered.	Methods, Section 2.1; Limitations.
	24b	Indicate where the review protocol can be accessed, or state that a protocol was not prepared.	Methods, Section 2.1.
	24c	Describe and explain any amendments to information provided at registration or in the protocol.	Not applicable.
Support	25	Describe sources of financial or non-financial support for the review, and the role of the funders or sponsors in the review.	Funding statement.
Competing interests	26	Declare any competing interests of review authors.	Conflict of Interest statement.

Section and Topic	Item #	Checklist item	Location where item is reported
Availability of data, code and other materials	27	Report which of the following are publicly available and where they can be found: template data collection forms; data extracted from included studies; data used for all analyses; analytic code; any other materials used in the review.	Data Availability statement; Table 2.

From: Page MJ, McKenzie JE, Bossuyt PM, Boutron I, Hoffmann TC, Mulrow CD, et al. The PRISMA 2020 statement: an updated guideline for reporting systematic reviews. BMJ 2021;372:n71. doi: 10.1136/bmj.n71. This work is licensed under CC BY 4.0. To view a copy of this license, visit https://creativecommons.org/licenses/by/4.0/

© 2026 The Author(s). Published by Bentham Science Publisher.

This is an open access article distributed under the terms of the Creative Commons Attribution 4.0 International Public License (CC-BY 4.0), a copy of which is available at: https://creativecommons.org/licenses/by/4.0/legalcode. This license permits unrestricted use, distribution, and reproduction in any medium, provided the original author and source are credited.

HOW TO CITE:
Yu C, Zhang H, Deng H. Diagnostic Performance of Ultrasonography and Mammography in Detecting Local Recurrence after Breast-Conserving Surgery: A Systematic Review and Exploratory Diagnostic Meta-analysis. Curr Med Imaging, 2026; 22: e15734056484401.
http://dx.doi.org/10.2174/0115734056484401260820095303
